# Comorbidity index for predicting mortality at 6 months after reperfusion therapy

**DOI:** 10.1038/s41598-021-85390-4

**Published:** 2021-03-16

**Authors:** Hyo Suk Nam, Young Dae Kim, Joonsang Yoo, Hyungjong Park, Byung Moon Kim, Oh Young Bang, Hyeon Chang Kim, Euna Han, Dong Joon Kim, Joonyung Heo, Minyoung Kim, Jin Kyo Choi, Kyung-Yul Lee, Hye Sun Lee, Dong Hoon Shin, Hye-Yeon Choi, Sung-Il Sohn, Jeong-Ho Hong, Jong Yun Lee, Jang-Hyun Baek, Gyu Sik Kim, Woo-Keun Seo, Jong-Won Chung, Seo Hyun Kim, Tae-Jin Song, Sang Won Han, Joong Hyun Park, Jinkwon Kim, Yo Han Jung, Han-Jin Cho, Seong Hwan Ahn, Sung Ik Lee, Kwon-Duk Seo, Ji Hoe Heo

**Affiliations:** 1 Department of Neurology, Yonsei University College of Medicine, 50-1 Yonsei-ro, Seodaemoon-gu, Seoul, 03722 Korea; 2 Department of Radiology, Yonsei University College of Medicine, Seoul, Korea; 3 Department of Neurology, Samsung Medical Center, Sungkyunkwan University School of Medicine, Seoul, Korea; 4 Department of Preventive Medicine, Yonsei University College of Medicine, Seoul, Korea; 5 College of Pharmacy, Yonsei Institute for Pharmaceutical Research, Yonsei University, Incheon, South Korea; 6 Department of Neurology, Gangnam Severance Hospital, Severance Institute for Vascular and Metabolic Research, Yonsei University College of Medicine, Seoul, Korea; 7 Department of Research Affairs, Biostatistics Collaboration Unit, Yonsei University College of Medicine, Seoul, Korea; 8 Department of Neurology, Gachon University Gil Medical Center, Incheon, Korea; 9 Department of Neurology, Kyung Hee University Hospital at Gangdong, Kyung Hee University School of Medicine, Seoul, Korea; 10 Department of Neurology, Brain Research Institute, Keimyung University School of Medicine, Daegu, Korea; 11 Department of Neurology, National Medical Center, Seoul, Korea; 12 Department of Neurology, Kangbuk Samsung Hospital, Sungkyunkwan University School of Medicine, Seoul, Korea; 13 Department of Neurology, National Health Insurance Service Ilsan Hospital, Ilsan, Korea; 14 Department of Neurology, Yonsei University Wonju College of Medicine, Wonju, Korea; 15 Department of Neurology, College of Medicine, Ewha Woman’s University, Seoul, Korea; 16 Department of Neurology, Sanggye Paik Hospital, Inje University College of Medicine, Seoul, Korea; 17 Department of Neurology, CHA Bundang Medical Center, CHA University, Seongnam, Korea; 18 Department of Neurology, Changwon Fatima Hospital, Changwon, Korea; 19 Department of Neurology, Pusan National University School of Medicine, Busan, Korea; 20 Department of Neurology, Chosun University School of Medicine, Gwangju, Korea; 21 Department of Neurology, Sanbon Hospital, Wonkwang University School of Medicine, Sanbon, Korea; 22 Department of Neurology, Yongin Severance Hospital, Yongin, Korea

**Keywords:** Cerebrovascular disorders, Stroke

## Abstract

The eligibility of reperfusion therapy has been expanded to increase the number of patients. However, it remains unclear the reperfusion therapy will be beneficial in stroke patients with various comorbidities. We developed a reperfusion comorbidity index for predicting 6-month mortality in patients with acute stroke receiving reperfusion therapy. The 19 comorbidities included in the Charlson comorbidity index were adopted and modified. We developed a statistical model and it was validated using data from a prospective cohort. Among 1026 patients in the retrospective nationwide reperfusion therapy registry, 845 (82.3%) had at least one comorbidity. As the number of comorbidities increased, the likelihood of mortality within 6 months also increased (*p* < 0.001). Six out of the 19 comorbidities were included for developing the reperfusion comorbidity index on the basis of the odds ratios in the multivariate logistic regression analysis. This index showed good prediction of 6-month mortality in the retrospective cohort (area under the curve [AUC], 0.747; 95% CI, 0.704–0.790) and in 333 patients in the prospective cohort (AUC, 0.784; 95% CI, 0.709–0.859). Consideration of comorbidities might be helpful for the prediction of the 6-month mortality in patients with acute ischemic stroke who receive reperfusion therapy.

## Introduction

The eligibility for reperfusion therapy has been expanded to increase the number of patients who would benefit from this treatment^1,2^. However, the benefit and risk should be considered in screening candidates for reperfusion therapy. Comorbidities are frequently found in patients with stroke and may be associated with patient outcomes, including mortality^3,4^. In the recent American Heart Association/American Stroke Association guidelines, some recommendations have been made on several individual comorbidities, such as diabetes mellitus, myocardial infarction, other cardiac diseases, systemic malignancy, end-stage renal disease, dementia, and sickle cell disease^5^. However, the levels of evidence are low. In addition, whether reperfusion therapy is beneficial in stroke patients with various comorbidities remains unclear.

The Charlson comorbidity index (CCI) is widely used in predicting 1-year mortality in various medical conditions and includes 19 comorbidities^6^. We developed a reperfusion comorbidity index primarily based on the comorbidities included in the CCI in patients with acute ischemic stroke receiving reperfusion therapy. We investigated its predictive ability for mortality within 6 months after reperfusion therapy.

## Results

### Study population

**Figure 1:**
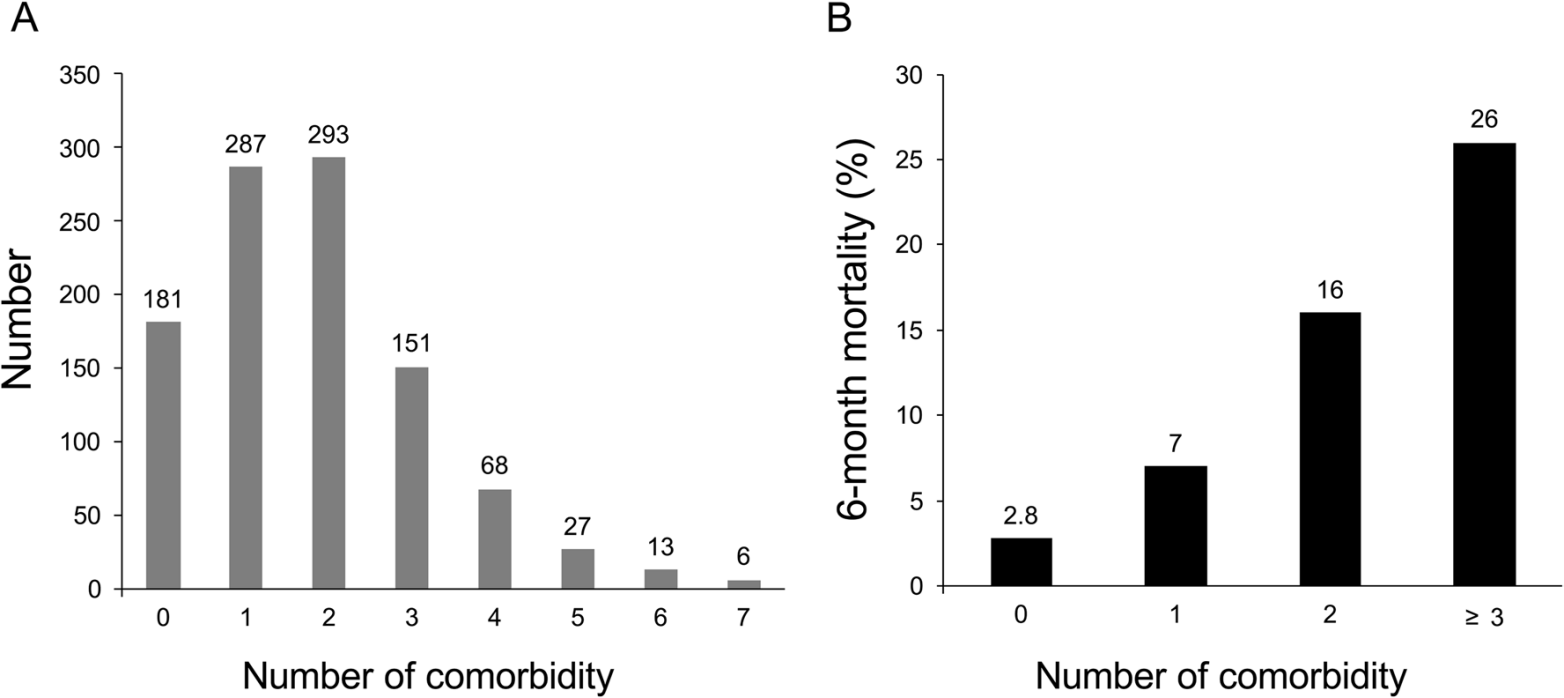
Frequency (**A**) and association of the number of comorbidities with 6-month mortality (**B**).

A total of 1026 patients were included in the retrospective cohort. Their median age was 71 (IQR, 62–76) years, and 589 patients (57.4%) were men. The median NIHSS score was 12.5 (IQR, 7–18) (Table 1). Of the 1026 enrolled patients, 845 (82.3%) had at least one comorbidity (median, 2 [IQR, 1–3]) (Fig. 1A). The most common comorbidity was atrial fibrillation (in 523 patients [51%]), followed by diabetes mellitus (in 432 patients [42.4%]), anemia (in 270 patients [26.3%]), and previous stroke (in 207 patients [20.2%]).

**Table 1:** Factors associated with mortality within 6 months.

	Death within 6 month (+)	Death within 6 month (−)	Total	*p*
	(N = 141)	(N = 885)	(N = 1026)	
Age	75.0 [69.0;80.0]	70.0 [62.0;76.0]	71.0 [62.0;76.0]	< 0.001
Sex				0.415
Men	76 (53.9)	513 (58.0)	589 (57.4)	
Women	65 (46.1)	372 (42.0)	437 (42.6)	
Hypertension	100 (70.9)	632 (71.4)	732 (71.3)	0.985
Hypercholesterolemia	49 (34.8)	272 (30.7)	321 (31.3)	0.391
Current smoking	19 (13.5)	210 (23.7)	229 (22.3)	0.009
Coronary artery disease	36 (25.5)	151 (17.1)	187 (18.2)	0.021
Valvular heart disease	6 (4.3)	34 (3.8)	40 (3.9)	0.999
Pre stroke mRS				0.001
0	107 (75.9)	791 (89.4)	898 (87.5)	
1	15 (10.6)	38 (4.3)	53 (5.2)	
2	7 (5.0)	20 (2.3)	27 (2.6)	
3	7 (5.0)	22 (2.5)	29 (2.8)	
4	4 (2.8)	9 (1.0)	13 (1.3)	
5	1 (0.7)	5 (0.6)	6 (0.6)	
Initial NIHSS score	17.0 [13.0;22.0]	12.0 [7.0;17.0]	12.5 [7.0;18.0]	< 0.001
Modality of reperfusion therapy				< 0.001
IV tPA	43 (30.5)	501 (56.6)	544 (53)	
IA UK	5 (3.5)	16 (1.8)	21 (2)	
EVT	49 (34.8)	166 (18.8)	215 (21)	
Combined treatment	44 (31.2)	202 (22.8)	246 (24)	
Myocardial infarction	16 (11.3)	45 (5.1)	61 (5.9)	0.006
Congestive heart failure	18 (12.8)	47 (5.3)	65 (6.3)	0.001
Peripheral artery obstructive disease	9 (6.4)	16 (1.8)	25 (2.4)	0.003
Previous stroke	39 (27.7)	168 (19.0)	207 (20.2)	0.023
Atrial fibrillation	84 (59.6)	439 (49.6)	523 (51.0)	0.035
Dementia	7 (5.0)	33 (3.7)	40 (3.9)	0.638
Depression	3 (2.1)	23 (2.6)	26 (2.5)	0.966
Pulmonary disease	7 (5.0)	33 (3.7)	40 (3.9)	0.638
Ulcer disease	12 (8.5)	21 (2.4)	33 (3.2)	< 0.001
Mild liver disease	6 (4.3)	17 (1.9)	23 (2.2)	0.152
Moderate to severe renal disease	16 (11.3)	40 (4.5)	56 (5.5)	0.002
Connective tissue disease	0 (0.0)	6 (0.7)	6 (0.6)	0.7
Diabetes	93 (66.0)	342 (38.6)	435 (42.4)	< 0.001
Anemia	61 (43.3)	209 (23.6)	270 (26.3)	< 0.001
AIDS	0 (0.0)	1 (0.1)	1 (0.1)	1
Cancer	5 (3.5)	19 (2.1)	24 (2.3)	0.471
Leukemia	0 (0)	0 (0)	0 (0)	NA
Lymphoma	3 (2.1)	1 (0.1)	4 (0.4)	0.005
Metastatic cancer	10 (7.1)	4 (0.5)	14 (1.4)	< 0.001
Number of comorbidity	2.0 [2.0; 3.0]	1.0 [1.0; 2.0]	2.0 [1.0; 3.0]	< 0.001

mRS, modified Rankin scale; NIHSS, National Institutes of Health Stroke Scale score; IV tPA, intravenous tissue-type plasminogen activator; IA UK, intraarterial urokinase; EVT, endovascular treatment; AIDS, Acquired Immune Deficiency Syndrome.

The prospective cohort included a total of 333 patients. Their median age was 71 (IQR, 59–78) years, and 208 patients (62.5%) were men. The median NIHSS score was 11 (IQR, 5–16). The demographic characteristics were similar between the retrospective and prospective cohorts, except for the presence/history of hypercholesterolemia, myocardial infarction, and metastatic cancer; current smoking status; initial NIHSS score; and treatment modalities (Supplemental Table 1).

### Association of comorbidity with 3-month modified Rankin Scale (mRS)

Among 1026 patients who enrolled in the retrospective cohorts, 3-month mRS was unavailable in 22 patients. Of the 1004 patients, univariable and multivariate analyses showed that poor functional outcome at 3 months (mRS ≥ 3) was associated with age, pre stroke mRS, dementia, and diabetes. When we further included initial NIHSS score, multivariable analysis revealed that age, pre stroke mRS, initial NIHSS score, dementia, diabetes, and anemia were independent predictors of poor functional outcome at 3 months (Supplemental Tables 3 and 4).

### Association of comorbidity with 6-month mortality

Among the 1026 patients, 141 (13.7%) died within 6 months. The median number of days until mortality was 12 (IQR, 3–76.5). The direct causes of mortality were cerebral infarction in 59 patients (41.8%), infection in 18 (12.8%), life-threatening hemorrhage, including intracranial hemorrhage in 17 (12.1%), cancer-related mortality in 10 (7.1%), myocardial infarction in 7 (0.5%), cardiac or pulmonary disease in 7 (0.5%), gastrointestinal hemorrhage in 1 (0.1%), and unknown in 22 (15.6%). Ten out of 14 patients (71.4%) with metastatic cancer died within 6 months (Table 1). As the number of comorbidities increased, the likelihood of mortality within 6 months also increased (2.8% in the patients without comorbidity, 7% in those with one comorbidity, 16% in those with two comorbidities, and 26% in those with three or more comorbidities; *p* < 0.001) (Fig. 1B).

### Prognostic model for predicting 6-month mortality

**Figure 2:**
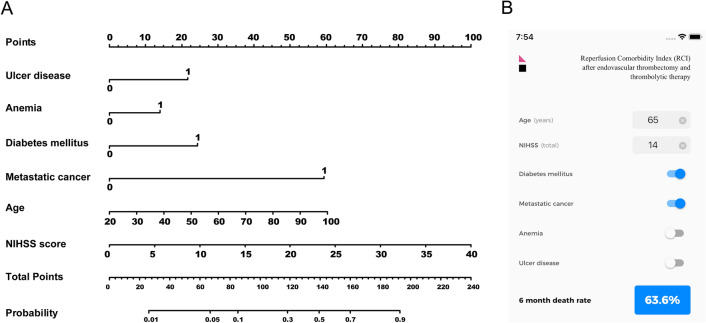
Nomogram (**A**) predicting mortality within 6 months and smartphone application (**B**). NIHSS, National Institutes of Health Stroke Scale score.

We developed the prognostic model for predicting 6-month mortality using data from the retrospective cohort. For the graphical computation, we created the nomogram. We also developed the smartphone application for fast and easy decision-making in clinical practice (Fig. 2). The prognostic model was validated in the retrospective cohort using data from the prospective cohort.

#### The reperfusion comorbidity index without considering initial stroke severity

The multivariate logistic regression analysis showed that age, myocardial infarction, congestive heart failure, ulcer disease, diabetes mellitus, anemia, and metastatic cancer were independently associated with 6-month mortality (Table 2). These variables were used to construct the model of the reperfusion comorbidity index for predicting 6-month mortality. The predicted probability of mortality within 6 months without considering initial stroke severity was calculated using the following equation: predicted probability = 1/(1 + exp [− A]), where A = − 5.2999 + 0.0357 × age + 0.6666 × myocardial infarction + 0.6622 × congestive heart failure + 1.0187 × ulcer disease + 1.0201 × diabetes mellitus + 0.5568 × anemia + 2.8128 × metastatic cancer.

**Table 2:** Univariable and multivariable analyses for the mortality within 6 months without considering initial stroke severity.

	Univariable		Multivariable	
	OR (95% CI)	*p* value	OR (95% CI)	*p* value
Age	1.042 (1.023–1.060)	< 0.0001	1.036 (1.017–1.057)	0.0003
Sex (women)	1.179 (0.825–1.685)	0.3649		
Myocardial infarction	2.389 (1.311–4.357)	0.0045	1.948 (1.010–3.755)	0.0465
Congestive heart failure	2.609 (1.468–4.639)	0.0011	1.939 (1.029–3.654)	0.0405
Peripheral artery obstructive disease	3.703 (1.604–8.552)	0.0022		
Previous stroke	1.632 (1.088–2.448)	0.0179		
Atrial fibrillation	1.497 (1.043–2.148)	0.0286		
Dementia	1.349 (0.585–3.111)	0.4824		
Depression	0.815 (0.241–2.750)	0.7414		
Pulmonary disease	1.349 (0.585–3.111)	0.4824		
Ulcerative disease	3.827 (1.839–7.966)	0.0003	2.770 (1.211–6.333)	0.0158
Liver disease	2.269 (0.879–5.857)	0.0903		
Renal disease	2.704 (1.470–4.974)	0.0014		
Connective tissue disease	0.479 (0.021–10.735)	0.6427		
Diabetes	3.076 (2.117–4.470)	< .0001	2.774 (1.877–4.099)	< .0001
Anemia	2.466 (1.708–3.562)	< .0001	1.745 (1.171–2.601)	0.0062
Cancer	1.659 (0.857–3.211)	0.1332		
Metastatic cancer	14.874 (5.555–39.825)	< .0001	16.656 (4.558–60.872)	< .0001

OR, odds ratio.

**Figure 3:**
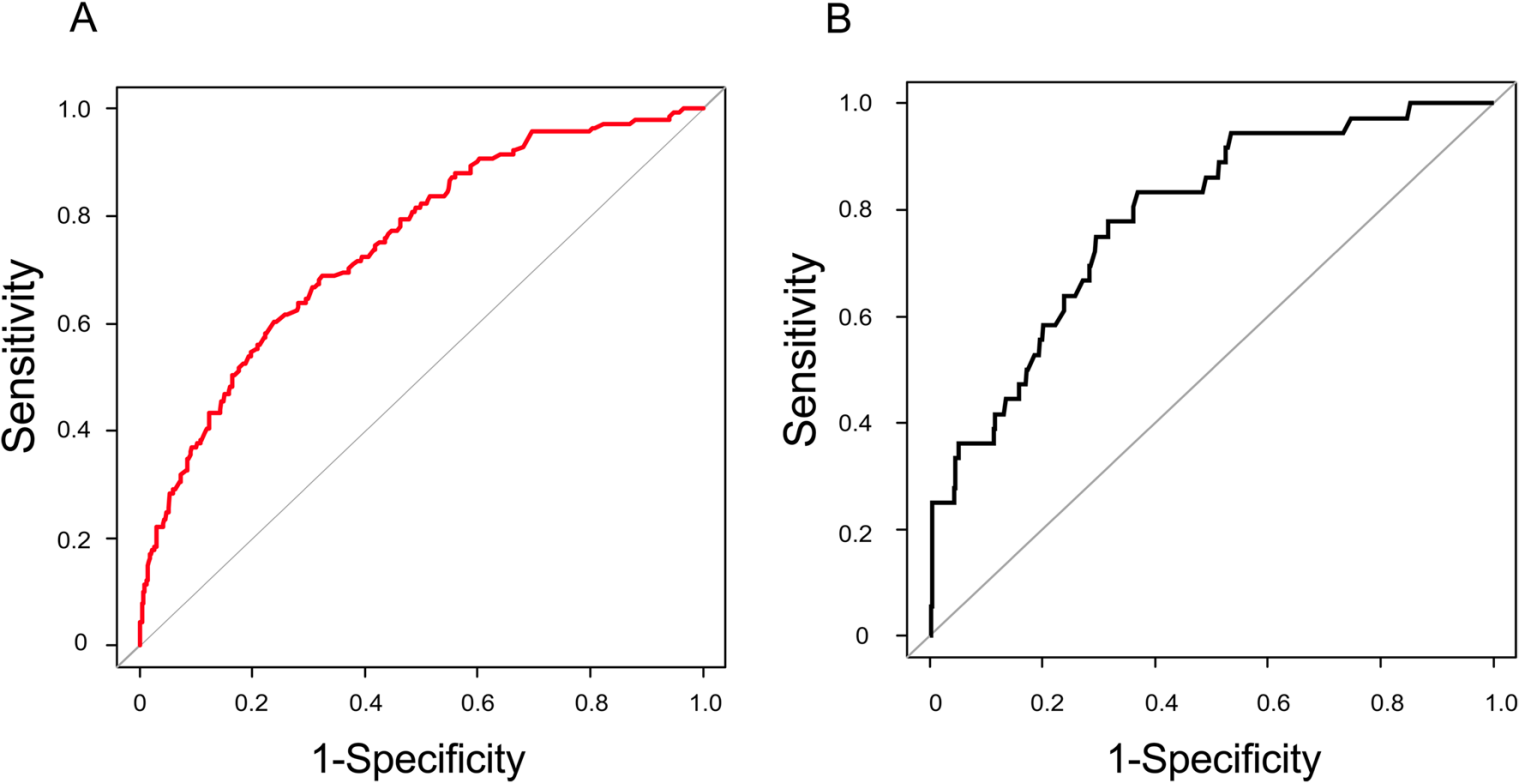
Internal validation (**A**) and external validation (**B**). The predictive ability of the reperfusion comorbidity index for 6-month mortality showed an area under the curve (AUC) of 0.747 (95% CI, 0.704–0.790) in retrospective cohort (**A**). In the prospective cohort, the predictive ability of the reperfusion comorbidity index was AUC of 0.784 (95% CI, 0.709–0.859) (**B**).

**Figure 4:**
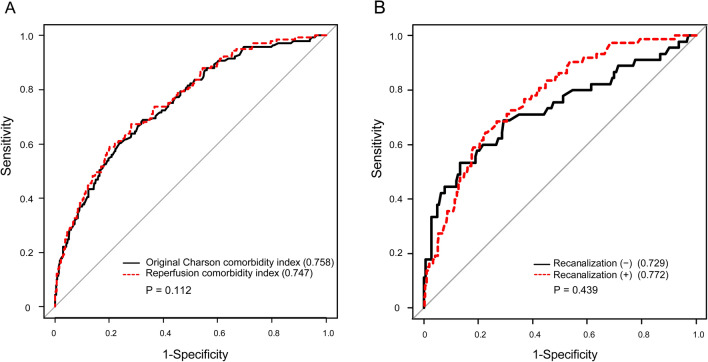
Receiver-operating characteristic curve (ROC) comparison death within 6 months mortality according to two models (**A**) and achievement of recanalization (**B**). ROC comparison for death within 6 months showed that the area under the curve (AUC) did `not differ between reperfusion comorbidity index (age + 6 comorbidities [myocardial infarction, congestive heart failure, ulcer disease, diabetes mellitus, anemia, and metastatic cancer]) and the original Charlson comorbidity index model (age + 16 comorbidities) (*p* = 0.112) (**A**). ROC curve comparison showed that the AUC did not differ according to recanalization status (*p* = 0.439) (**B**).

The predictive ability of the reperfusion comorbidity index for 6-month mortality showed an AUC of 0.747 (95% CI, 0.704–0.790) In the Hosmer and Lemeshow test, the *p* value was 0.786, indicating that the model was suitable (Fig. 3A). The calibration plots showed a close approximation to the logistic calibration of the nomogram. ROC comparison for death within 6 months showed that the AUC did not differ between reperfusion comorbidity index (age + 6 comorbidities [myocardial infarction, congestive heart failure, ulcer disease, diabetes mellitus, anemia, and metastatic cancer]) and the original CCI (age + 16 comorbidities) (0.747 [95% CI, 0.704–0.790] vs. 0.758 [95% CI, 0.716–0.800]; *p* = 0.112) (Fig. 4A). The AUC did not differ between the patients with and without successful recanalization (with recanalization, 0.772 [95% CI, 0.717–0.826] vs. without recanalization, 0.729 [95% CI, 0.634–0.824]; *p* = 0.439) (Fig. 4B).

In the prospective cohort, the predictive ability of the reperfusion comorbidity without considering initial stroke severity showed an AUC of 0.784 (95% CI, 0.709–0.859). In the Hosmer and Lemeshow test, the *p* value was 0.140, indicating that the model was suitable (Fig. 3B).

#### The reperfusion comorbidity index considering initial stroke severity

The multivariate logistic regression analysis showed that age, initial NIHSS score, ulcer disease, diabetes mellitus, anemia, and metastatic cancer were independently associated with 6-month mortality (Supplemental Table 5). The predicted probability of mortality within 6 months with considering initial stroke severity was calculated using the following equation: predicted probability = 1/(1 + exp  [− A]), where A = − 6.2807 + 0.0307 × age + 0.1019 × NIHSS score + 0.8817 × ulcer disease + 0.571 × anemia + 0.9953 × diabetes mellitus + 2.4194 × metastatic cancer. ROC comparison for death within 6 months showed that the AUC did not differ between the reperfusion comorbidity index considering initial stroke severity (age, NIHSS score + 4 comorbidities [ulcer disease, diabetes mellitus, anemia, and metastatic cancer]) and the original CCI (age, NIHSS score + 16 comorbidities) (0.781 [95% CI, 0.738–0.824] vs. 0.792 [95% CI, 0.751–0.832]; *p* = 0.120) (Supplemental Fig. 1A).

In the prospective cohort, the predictive ability of the reperfusion comorbidity index considering initial stroke severity showed an area under the curve AUC of 0.821 (95% CI, 0.759–0.884). In the Hosmer and Lemeshow test, the *p* value was 0.098, indicating that the model was suitable (Supplemental Fig. 1B).

## Discussion

In this study, we found that the patients who received reperfusion therapy frequently had comorbidities. Over 80% of them had one or more comorbidities. Mortality within 6 months was significantly associated with the number of comorbidities. In a previous study, the comorbidity burden was a strong prognostic factor for both short- and long-term mortalities in patients with stroke^4^. Comorbidities are closely related with each other and worsen the prognosis beyond that expected through the independent effects of each comorbid condition^4^. Comorbidities may also influence the clinical outcomes of stroke in terms of the diagnostic process, treatment effects, complication rates, and rehabilitation^4,7^.

We found that myocardial infarction, congestive heart failure, ulcer disease, diabetes mellitus, anemia, and metastatic cancer were independent predictors of mortality within 6 months. These comorbidities are known to be associated with an increased risk of mortality. Acute myocardial infarction complicating stroke accounts for 20% of in-hospital mortality cases. In-hospital mortality is three-fold higher in patients with stroke and acute myocardial infarction than in those without the latter^8,9^. Patients with stroke and congestive heart failure showed higher risks of mortality at 30 days (24.5%) and at 1 year (44.3%)^10,11^. Peptic ulcer disease was an independent risk factor for ischemic stroke and mortality^12^. Almost 12% of patients with peptic ulcer disease died within 1 year, and the main causes of mortality were cardiovascular disease and malignancy^13^. Admission blood glucose level and history of diabetes mellitus are strong predictors of poor outcomes^14–16^. Anemia on admission was also associated with an increased risk of mortality in ischemic stroke^17^. Cancer is unquestionably associated with the risk of mortality^14,18^. The presence of metastasis is particularly associated with the short-term risk of mortality. Approximately two-thirds of patients with stroke and metastatic cancer died within 6 months after stroke^19^.

There were slight differences of comorbidities between prediction of 6-month mortality and that of functional outcomes as 3 months. While metastatic cancer was a strong prognostic indicator for 6-month mortality, it was not associated with poor functional outcomes at 3 months. In contrast, dementia was associated with poor functional outcomes at 3 months, while it was not associated with 6-month mortality.

Herein, we included comorbidities based on the CCI. The CCI was developed as a prognostic indicator in patients with a variety of conditions admitted to a general medical service^6^. It has been also proven to be a valid parameter for predicting mortality within 1 year and functional outcomes in patients with stroke^3,4^. Patients with severe comorbidities were more likely to die during hospitalization^20^. We slightly modified the CCI by removing or replacing some comorbidities considering that our study population consisted of patients with acute stroke. We also examined mortality within 6 months because we wanted to investigate the role of the comorbidity index as a parameter for selecting patients for reperfusion therapy. The 6-month survival was suggested in the guidelines as a criterion for alteplase treatment in patients with stroke and systemic malignancy. This study showed that our comorbidity index model may be useful for predicting mortality within 6 months in patients with acute stroke who are potential candidates for reperfusion therapy.

We developed an additional model that includes NIHSS score to comorbidities because NIHSS is routinely determined and a strong prognostic factor in acute stroke. For the model, 6 or 4 (when including NIHSS score) out of the 19 comorbidities were included on the basis of the odds ratios in the multivariate logistic regression analysis. A simple prognostic tool should be used for screening candidates of reperfusion therapy in busy emergency rooms. Obtaining information on many individual comorbidities, such as those included in the CCI, is impractical in emergent conditions in relation to reperfusion therapy. The predictability of 6-month mortality of this models was similar to that of the original CCI model. Consideration of just 6 or 4 comorbidities instead of full comorbidities in the original CCI may be enough to predict mortality within 6 months in patients receiving reperfusion therapy. We also developed a nomogram and a smartphone application, which can be used easily and rapidly in busy emergency rooms^21^.

Many index scores for reperfusion therapy to predict stroke outcomes have already been proposed. For examples, those include the Pittsburgh Response to Endovascular Therapy (PRE) score^22^, the Stroke Prognostication Using Age and National Institutes of Health Stroke Scale (SPAN) index^23^, the Totaled Health Risks in Vascular Events (THRIVE) score^24^, the Houston Intra-Arterial Therapy (HIAT) score^25^, and the HIAT2 score^26^. We summarize a table of each scoring system and compared to our reperfusion comorbidity index (Supplemental table 6). The AUC value of our reperfusion comorbidity index was compatible with those of other scoring systems. Our comorbidity index is different from others in that it can be calculated before reperfusion therapy without further imaging analysis such as the Alberta stroke program early CT score (ASPECTS).

There are some limitations in this study. First, the study population was obtained from a nationwide registry, but in a single country with a single ethnicity. Comorbidities may be different among countries and ethnicities. Therefore, our model should be validated in other cohorts. Second, although a relatively large number of patients were included in this study, some comorbidities were not included in the final predictive model because of their very low prevalence. However, they may influence the outcomes. Although almost all necessary information on comorbidities were obtainable by thorough medical review, particularly in the prospective cohort. There still might be some comorbidities that were unobtainable due to various reasons. Third, we did not include some factors associated with the outcomes of the patients receiving reperfusion therapy for the predictive model (e.g. achievement of recanalization, infarction volume, and complication after reperfusion therapy, such as intracerebral hemorrhage). We wanted to develop a model predicting 6-month mortality, which can be available before reperfusion therapy. Finally, our comorbidity index provides probability of mortality within 6 months to guide and help for decision making of reperfusion therapy. However, quality of life is also very important. The decision regarding reperfusion therapy should be individualized and further research is required.

In conclusion, we developed and validated our simple reperfusion comorbidity index to determine the probability of mortality within 6 months using a large actual-clinical setting multicenter cohort in stroke patients receiving reperfusion therapy. This model may be helpful for prediction of 6-month mortality regarding reperfusion therapy in patients with acute ischemic stroke.

## Methods

### Patients

We analyzed data from the SElection CRiteria in Endovascular thrombectomy and Thrombolytic therapy (SECRET) registry. This nationwide registry included consecutive patients with hyperacute ischemic stroke who received intravenous thrombolysis or endovascular thrombectomy. The SECRET registry aimed at exploring the patient selection criteria for reperfusion therapy, especially in relation to clots, core, collaterals, and comorbidities. It included a retrospective cohort and a prospective cohort. The retrospective cohort included 1026 patients from 15 hospitals who received treatment between January 2012 and December 2015. The prospective cohort included 333 patients from 13 hospitals who received treatment between November 2016 and December 2017.

The SECRET registry collected the following data: (1) demographic and clinical data, (2) information on reperfusion therapy, (3) comorbidities, and (4) imaging data. We determined the modified Rankin scale score at 90 days, mortality within 6 months, and the cause of death. Recanalization was assessed using the modified treatment in cerebral infarction score (mTICI). Successful recanalization was defined as mTICI 2b or 3. Symptomatic intracerebral hemorrhage was defined in accordance with the European Cooperative Acute Stroke Study 3 criteria^2^.

### Comorbidities

We determined the presence of comorbidities based on the CCI, which is widely used to predict 1-year mortality^3,27^. In this study, we modified the CCI, considering patients with stroke as the study population. We omitted paralysis from the original CCI because it is a common symptom or sign of stroke; conversely, we added anemia^17^, atrial fibrillation^28^, and depression^29^ as comorbidities, as they are common and are known to be associated with patient outcomes. Further, we merged diabetes mellitus with and without end-organ damage as diabetes mellitus. Finally, 19 comorbidities were included: myocardial infarction, congestive heart failure, peripheral vascular disease, previous stroke, atrial fibrillation, dementia, depression, chronic pulmonary disease, ulcer disease, mild liver disease, moderate or severe renal disease, connective tissue disease or rheumatic disease, diabetes mellitus, anemia, acquired immune deficiency syndrome, non-metastatic solid tumor, leukemia, lymphoma, and metastatic cancer.

We collected full information on the 19 comorbidities after completion of the reperfusion therapy via history taking and the thorough review of medical records at each study hospital. Neurologists or trained research assistants (nurses) determined the presence of comorbidities according to the definition of a comorbidity (Supplemental material). The number of comorbidities was categorized as 0, 1, 2, or ≥ 3^4^.

### Statistical analysis

Data were expressed as means ± SDs, medians and interquartile ranges (IQRs), or frequencies (percentages), as appropriate. Variables were tested for normality using the Kolmogorov–Smirnov test. Differences between the groups were compared using an independent sample t-test or the Mann–Whitney U-test for continuous variables and the chi-square test for categorical variables.

We developed a statistical model to predict 6-month mortality using univariate and multivariate logistic regression analyses with backward (Wald) selection of predictors in the retrospective cohort (the reperfusion comorbidity index). A multivariate analysis was performed using all variables with *p* values of < 0.05 in the univariate analysis. Comorbidities including leukemia, lymphoma, AIDS with a low prevalence (n < 5) were excluded in the multivariate analysis model. Therefore, 16 comorbidities were analyzed. We also developed another statistical model that added the National Institutes of Health Stroke Scale (NIHSS) score to comorbidities. The models were validated using data from a prospective cohort.

We determined the area under the receiver-operating characteristic curve (ROC) to predict 6-month mortality. The Hosmer and Lemeshow goodness-of-fit test was used to assess the acceptability of the predictive models. This test also determined how well the nomogram was calibrated. Close approximation between the observed and predicted probabilities demonstrated good calibration and confirmed the exportability of the model. We compared the predictability of the reperfusion comorbidity index with that of the original CCI. We also compared the AUC between the patients with recanalization and those without successful recanalization.

The nomogram for predicting 6-month mortality was constructed on the basis of the multivariate models with data from the retrospective cohort. Validation was performed using the prospective cohort. We also developed iPhone and android smartphone applications. The applications calculated the probability of 6-month mortality upon selection of comorbidities.

All *p* values were two-sided. *p* values of < 0.05 were considered significant. Statistical analyses were performed using SAS version 9.3 (SAS Institute Inc., Cary, NC, USA) and R package version 3.1.2 (R Foundation for Statistical Computing, Vienna, Austria; http://www.R-project.org).

#### Standard protocol approval, registration, and patient consent

The study is registered to Clinicaltrials.gov (NCT02964052). The study and registry were approved by the institutional review board (Yonsei University College of Medicine, 4-2015-1196). All methods were performed in accordance with the relevant guidelines and regulations. For the prospectively enrolled patients, written informed consent was obtained from the patients themselves or their next of kin.

## Supplementary Information

Supplementary Information

## Data Availability

The datasets generated during and/or analysed during the current study are available from the corresponding author on reasonable request.
