# Supplementary material for: Comorbidity index for predicting mortality at 6 months after reperfusion therapy
Source: Sci Rep. 2021 Mar 16;11:5963. doi: 10.1038/s41598-021-85390-4 (PMC7966783; doi:10.1038/s41598-021-85390-4)
Supplement: Supplementary file 1 — Supplementary Information [file 41598_2021_85390_MOESM1_ESM.docx]

# SUPPLEMENTAL MATERIAL

# Comorbidity index for predicting mortality at 6 months after reperfusion therapy

Hyo Suk Nam, MD, PhD,^1^ Young Dae Kim, MD, PhD,^1^ Joonsang Yoo, MD,^1,13^ Hyungjong Park, MD,^1,10^ Byung Moon Kim, MD, PhD,^2^ Oh Young Bang, MD, PhD,^3^ Hyeon Chang Kim, MD, PhD,^4^ Euna Han, PhD,^5^ Dong Joon Kim, MD,^2^ PhD, Joonyung Heo, MD,^1^ Minyoung Kim, BS,^1^ Jin Kyo Choi, MD,^1^ Kyung-Yul Lee, MD,^6^ Hye Sun Lee, PhD,^7^ Dong Hoon Shin, MD, PhD,^8^ Hye-Yeon Choi, MD, PhD,^9^ Sung-Il Sohn, MD, PhD,^10^ Jeong-Ho Hong, MD, PhD,^10^ Jong Yun Lee, MD,^11^ Jang-Hyun Baek, MD,^11,12^ Gyu Sik Kim, MD,^13^ Woo-Keun Seo, MD, PhD,^3^ Jong-Won Chung, MD,^3^ Seo Hyun Kim, MD, PhD,^14^ Tae-Jin Song, MD, PhD,^15^ Sang Won Han, MD,^16^ Joong Hyun Park, MD,^16^ Jinkwon Kim, MD, PhD,^17,22^ Yo Han Jung, MD, PhD,^6,18^ Han-Jin Cho, MD, PhD ^,19^ Seong Hwan Ahn, MD,^20^ Sung Ik Lee, MD,^21^ Kwon-Duk Seo, MD,^13,21^ and Ji Hoe Heo, MD, PhD^1*^

^1^Department of Neurology, Yonsei University College of Medicine, Seoul, Korea

^2^Department of Radiology, Yonsei University College of Medicine, Seoul, Korea

^3^Department of Neurology, Samsung Medical Center, Sungkyunkwan University School of Medicine, Seoul, Korea,

^4^Department of Preventive Medicine, Yonsei University College of Medicine, Seoul, Korea

^5^College of Pharmacy, Yonsei Institute for Pharmaceutical Research, Yonsei University, Incheon, South Korea

^6^Department of Neurology, Gangnam Severance Hospital, Severance Institute for Vascular and Metabolic Research, Yonsei University College of Medicine, Seoul, Korea

^7^Department of Research Affairs, Biostatistics Collaboration Unit, Yonsei University College of Medicine, Seoul, Korea

^8^Department of Neurology, Gachon University Gil Medical Center, Incheon, Korea

^9^Department of Neurology, Kyung Hee University Hospital at Gangdong, Kyung Hee University School of Medicine, Seoul, Korea

^10^ Department of Neurology, Brain Research Institute, Keimyung University School of Medicine, Daegu, Korea

^11^Department of Neurology, National Medical Center, Seoul, Korea

^12^Department of Neurology, Kangbuk Samsung Hospital, Sungkyunkwan University School of Medicine, Seoul, Korea

^13^Department of Neurology National Health Insurance Service Ilsan Hospital, Ilsan, Korea

^14^Department of Neurology, Yonsei University Wonju College of Medicine, Wonju, Korea

^15^Department of Neurology, College of Medicine, Ewha Woman’s University, Seoul, Korea

^16^Department of Neurology, Sanggye Paik Hospital, Inje University College of Medicine, Seoul, Korea

^17^Department of Neurology, CHA Bundang Medical Center, CHA University, Seongnam, Korea

^18^Department of Neurology, Changwon Fatima Hospital, Changwon, Korea

^19^ Department of Neurology, Pusan National University School of Medicine, Busan, Korea

^20^Department of Neurology, Chosun University School of Medicine, Gwangju, Korea

^21^Department of Neurology, Sanbon Hospital, Wonkwang University School of Medicine, Sanbon, Korea

^22^Department of Neurology, Yongin Severance Hospital, Yongin, Korea

***Corresponding author:**

Ji Hoe Heo, MD, PhD

Department of Neurology, Yonsei University College of Medicine

50-1 Yonsei-ro, Seodaemoon-gu, Seoul 03722, Korea

Tel: 82-2-2228-1605, Fax: 82-2-393-0705

E-mail: jhheo@yuhs.ac

# Supplemental Table 1. Comparison between retrospective and prospective cohorts

|  | Retrospective cohort | Prospective cohort | Total | p |
| --- | --- | --- | --- | --- |
|  | (N=1026) | (N=333) | (N=1359) |  |
| Age | 71.0 [62.0;76.0] | 71.0 [59.0;78.0] | 71.0 [61.0;77.0] | 0.741 |
| Sex |  |  |  | 0.118 |
| Men | 589 (57.4) | 208 (62.5) | 797 (58.6) |  |
| Women | 437 (42.6) | 125 (37.5) | 562 (41.4) |  |
| Hypertension | 732 (71.3) | 237 (71.2) | 969 (71.3) | 1 |
| Hypercholesterolemia | 321 (31.3) | 142 (42.6) | 463 (34.1) | < 0.001 |
| Current smoking | 229 (22.3) | 74 (22.2) | 303 (22.3) | 1 |
| Coronary artery disease | 187 (18.2) | 58 (17.4) | 245 (18.0) | 0.801 |
| Valvular heart disease | 40 (3.9) | 13 (3.9) | 53 (3.9) | 1 |
| Pre stroke mRS score |  |  |  | 0.096 |
| 0 | 898 (87.5) | 309 (92.8) | 1207 (88.8) |  |
| 1 | 53 (5.2) | 11 (3.3) | 64 (4.7) |  |
| 2 | 27 (2.6) | 4 (1.2) | 31 (2.3) |  |
| 3 | 29 (2.8) | 3 (0.9) | 32 (2.4) |  |
| 4 | 13 (1.3) | 5 (1.5) | 18 (1.3) |  |
| 5 | 6 (0.6) | 1 (0.3) | 7 (0.5) |  |
| Modality of reperfusion therapy |  |  |  | < 0.001 |
| IV tPA | 544 (53.0) | 109 (32.7) | 653 (48.1) |  |
| Endovascular thrombectomy | 215 (21.0) | 114 (34.2) | 329 (24.2) |  |
| IAUK | 21 (2.0) | 0 (0) | 21 (1.5) |  |
| Combined treatment | 246 (24.0) | 110 (33.0) | 356 (26.2) |  |
| Initial NIHSS score | 12.5 [ 7.0;18.0] | 11.0 [ 5.0;16.0] | 12.0 [ 7.0;17.0] | 0.002 |
| Myocardial infarction | 61 (5.9) | 9 (2.7) | 70 (5.2) | 0.029 |
| Congestive heart failure | 65 (6.3) | 13 (3.9) | 78 (5.7) | 0.128 |
| Peripheral artery obstructive disease | 25 (2.4) | 5 (1.5) | 30 (2.2) | 0.427 |
| Previous stroke | 207 (20.2) | 70 (21.0) | 277 (20.4) | 0.799 |
| Atrial fibrillation | 523 (51.0) | 137 (41.1) | 660 (48.6) | 0.002 |
| Dementia | 40 (3.9) | 16 (4.8) | 56 (4.1) | 0.573 |
| Depression | 26 (2.5) | 8 (2.4) | 34 (2.5) | 1 |
| Pulmonary disease | 40 (3.9) | 13 (3.9) | 53 (3.9) | 1 |
| Ulcer disease | 33 (3.2) | 8 (2.4) | 41 (3.0) | 0.569 |
| Mild liver disease | 23 (2.2) | 10 (3.0) | 33 (2.4) | 0.562 |
| Moderate to severe renal disease | 56 (5.5) | 16 (4.8) | 72 (5.3) | 0.748 |
| Connective tissue disease | 6 (0.6) | 6 (1.8) | 12 (0.9) | 0.084 |
| Diabetes | 435 (42.4) | 144 (43.2) | 579 (42.6) | 0.836 |
| Anemia | 270 (26.3) | 84 (25.2) | 354 (26.0) | 0.747 |
| AIDS | 1 (0.1) | 0 (0.0) | 1 (0.1) | 1 |
| Cancer | 24 (2.3) | 3 (0.9) | 27 (2.0) | 0.159 |
| Leukemia | 0 (0.0) | 1 (0.3) | 1 (0.1) | 0.553 |
| Lymphoma | 4 (0.4) | 0 (0.0) | 4 (0.3) | 0.576 |
| Metastatic cancer | 14 (1.4) | 13 ( 3.9) | 27 (2.0) | 0.008 |

mRS, modified Rankin scale; tPA, tissue-type plasminogen activator; IA UK, intraarterial urokinase; NIHSS, National Institutes of Health Stroke Scale.

# Supplemental Table 2. Univariable analysis for factors associated with poor outcome (mRS ≥3) at 3 months

|  | Poor outcome | Good outcome | Total | p |
| --- | --- | --- | --- | --- |
|  | (N=457) | (N=547) | (N=1004) |  |
| Age | 71.3 ± 11.2 | 66.4 ± 11.4 | 68.7 ± 11.5 | < 0.001 |
| Sex |  |  |  | 0.024 |
| Men | 245 (53.6%) | 333 (60.9%) | 578 (57.6%) |  |
| Women | 212 (46.4%) | 214 (39.1%) | 426 (42.4%) |  |
| Hypertension | 343 (75.1%) | 371 (67.8%) | 714 (71.1%) | 0.014 |
| Hypercholesterolemia | 154 (33.7%) | 164 (30.0%) | 318 (31.7%) | 0.233 |
| Current smoking | 85 (18.6%) | 143 (26.1%) | 228 (22.7%) | 0.006 |
| Coronary artery disease | 82 (17.9%) | 101 (18.5%) | 183 (18.2%) | 0.896 |
| Valvular heart disease | 15 ( 3.3%) | 24 ( 4.4%) | 39 ( 3.9%) | 0.46 |
| Pre stroke mRS |  |  |  | < 0.001 |
| 0 | 355 (77.7%) | 526 (96.2%) | 881 (87.7%) |  |
| 1 | 39 ( 8.5%) | 12 ( 2.2%) | 51 ( 5.1%) |  |
| 2 | 21 ( 4.6%) | 4 ( 0.7%) | 25 ( 2.5%) |  |
| 3 | 25 ( 5.5%) | 4 ( 0.7%) | 29 ( 2.9%) |  |
| 4 | 11 ( 2.4%) | 1 ( 0.2%) | 12 ( 1.2%) |  |
| 5 | 6 ( 1.3%) | 0 ( 0.0%) | 6 ( 0.6%) |  |
| Initial NIHSS | 15.4 ± 6.2 | 10.6 ± 5.6 | 12.7 ± 6.4 | < 0.001 |
| Modality of reperfusion therapy |  |  |  | < 0.001 |
| IV tPA | 193 (42.2%) | 332 (60.7%) | 525 (52.3%) |  |
| IA UK | 14 ( 3.1%) | 7 ( 1.3%) | 21 ( 2.1%) |  |
| EVT | 125 (27.4%) | 89 (16.3%) | 214 (21.3%) |  |
| Combined | 125 (27.4%) | 119 (21.8%) | 244 (24.3%) |  |
| Myocardial infarction | 33 ( 7.2%) | 27 ( 4.9%) | 60 ( 6.0%) | 0.165 |
| Congestive heart failure | 35 ( 7.7%) | 27 ( 4.9%) | 62 ( 6.2%) | 0.098 |
| Peripheral artery obstructive disease | 15 ( 3.3%) | 10 ( 1.8%) | 25 ( 2.5%) | 0.204 |
| Previous stroke | 119 (26.0%) | 85 (15.5%) | 204 (20.3%) | < 0.001 |
| Atrial fibrillation | 252 (55.1%) | 263 (48.1%) | 515 (51.3%) | 0.03 |
| Dementia | 33 ( 7.2%) | 5 ( 0.9%) | 38 ( 3.8%) | < 0.001 |
| Depression | 14 ( 3.1%) | 11 ( 2.0%) | 25 ( 2.5%) | 0.388 |
| Pulmonary disease | 25 ( 5.5%) | 13 ( 2.4%) | 38 ( 3.8%) | 0.017 |
| Ulcer disease | 23 ( 5.0%) | 9 ( 1.6%) | 32 ( 3.2%) | 0.004 |
| Mild liver disease | 12 ( 2.6%) | 11 ( 2.0%) | 23 ( 2.3%) | 0.662 |
| Moderate to severe renal disease | 34 ( 7.4%) | 19 ( 3.5%) | 53 ( 5.3%) | 0.008 |
| Connective tissue disease | 2 ( 0.4%) | 3 ( 0.5%) | 5 ( 0.5%) | 1 |
| Diabetes | 250 (54.7%) | 175 (32.0%) | 425 (42.3%) | < 0.001 |
| Anemia | 152 (33.3%) | 110 (20.1%) | 262 (26.1%) | < 0.001 |
| AIDS | 0 ( 0.0%) | 1 ( 0.2%) | 1 ( 0.1%) | 1 |
| Cancer | 11 ( 2.4%) | 13 ( 2.4%) | 24 ( 2.4%) | 1 |
| Leukemia | 0 (0%) | 0 (0%) | 0 (0%) | NA |
| Lymphoma | 4 ( 0.9%) | 0 ( 0.0%) | 4 ( 0.4%) | 0.091 |
| Metastatic cancer | 11 ( 2.4%) | 3 ( 0.5%) | 14 ( 1.4%) | 0.026 |

mRS, modified Rankin scale; NIHSS, National Institutes of Health Stroke Scale score; IV tPA, intravenous tissue-type plasminogen activator; IA UK, intraarterial urokinase; EVT, endovascular thrombectomy; TICI, thrombolysis in cerebral infarction; AIDS, Acquired Immune Deficiency Syndrome.

# Supplemental Table 3. Multivariable analysis for factors associated with poor outcome (mRS ≥3) at 3 months without considering initial stroke severity

|  | B | S.E. | Wald | p | Exp(B) |
| --- | --- | --- | --- | --- | --- |
| Age | 0.03 | 0.007 | 17.807 | <0.001 | 1.031 (1.016-1.046) |
| Sex | 0.057 | 0.157 | 0.131 | 0.717 | 1.059 (0.778-1.441) |
| Hypertension | -0.133 | 0.166 | 0.639 | 0.424 | 0.876 (0.632-1.212) |
| Current smoking | 0.132 | 0.191 | 0.482 | 0.487 | 1.142 (0.786-1.659) |
| Pre stroke mRS | 0.817 | 0.137 | 35.419 | <0.001 | 2.263 (1.729-2.961) |
| Previous stroke | 0.246 | 0.183 | 1.794 | 0.18 | 1.278 (0.892-1.831) |
| Atrial fibrillation | 0.024 | 0.146 | 0.027 | 0.87 | 1.024 (0.769-1.365) |
| Dementia | 1.578 | 0.516 | 9.335 | 0.002 | 4.843 (1.760-13.323) |
| Pulmonary disease | 0.479 | 0.391 | 1.503 | 0.22 | 1.614 (0.751-3.470) |
| Ulcer disease | 0.785 | 0.443 | 3.141 | 0.076 | 2.193 (0.920-5.226) |
| Moderate to severe renal disease | 0.37 | 0.335 | 1.217 | 0.27 | 1.447 (0.750-2.790) |
| Anemia | 0.296 | 0.168 | 3.118 | 0.077 | 1.344 (0.968-1.867) |
| Diabetes | 0.956 | 0.144 | 44.12 | <0.001 | 2.602 (1.962-3.451) |
| Metastatic cancer | 0.992 | 0.716 | 1.923 | 0.166 | 2.698 (0.663-10.971) |

# Supplemental Table 4. Multivariable analysis for factors associated with poor outcome (mRS ≥3) at 3 months with considering initial stroke severity

|  | B | S.E. | Wald | p | Exp(B) |
| --- | --- | --- | --- | --- | --- |
| Age | 0.028 | 0.008 | 13.207 | <0.001 | 1.028 (1.013-1.044) |
| Sex | -0.104 | 0.17 | 0.374 | 0.541 | 0.901 (0.645-1.258) |
| Hypertension | -0.061 | 0.178 | 0.12 | 0.729 | 0.94 (0.664-1.332) |
| Current smoking | 0.228 | 0.202 | 1.282 | 0.257 | 1.257 (0.846-1.866) |
| Pre stroke mRS | 0.801 | 0.147 | 29.754 | <0.001 | 2.227 (1.670-2.970) |
| Initial NIHSS score | 0.135 | 0.014 | 98.665 | <0.001 | 1.145 (1.115-1.176) |
| Previous stroke | 0.185 | 0.196 | 0.892 | 0.345 | 1.204 (0.819-1.769) |
| Atrial fibrillation | -0.314 | 0.16 | 3.841 | 0.05 | 0.73 (0.533-1.000) |
| Dementia | 1.75 | 0.546 | 10.268 | 0.001 | 5.756 (1.973-16.789) |
| Pulmonary disease | 0.682 | 0.417 | 2.671 | 0.102 | 1.978 (0.873-4.481) |
| Ulcer disease | 0.66 | 0.491 | 1.807 | 0.179 | 1.935 (0.739-5.067) |
| Moderate to severe renal disease | 0.384 | 0.349 | 1.211 | 0.271 | 1.469 (0.741-2.912) |
| Anemia | 0.357 | 0.179 | 3.996 | 0.046 | 1.429 (1.007-2.028) |
| Diabetes | 1.014 | 0.155 | 42.891 | <0.001 | 2.755 (2.034-3.732) |
| Metastatic cancer | 0.323 | 0.753 | 0.184 | 0.668 | 1.381 (0.316-6.042) |

# Supplemental Table 5. Univariable and multivariable analyses for the mortality within 6 months with considering initial stroke severity

| Variable | Univariable | | | Multivariable | |
| --- | --- | --- | --- | --- | --- |
|  | OR(95% CI) | p-value | OR(95% CI) | | p-value |
| Age | 1.042 (1.023-1.060) | <0.0001 | 1.031(1.011-1.051) | | 0.002 |
| Sex (women) | 1.179 (0.825-1.685) | 0.3649 |  | |  |
| NIHSS score | 1.125 (1.092-1.159) | <0.0001 | 1.107 (1.073-1.142) | | <0.0001 |
| Myocardial infarction | 2.389 (1.311-4.357) | 0.0045 |  | |  |
| Congestive heart failure | 2.609 (1.468-4.639) | 0.0011 |  | |  |
| Peripheral artery obstructive disease | 3.703 (1.604-8.552) | 0.0022 |  | |  |
| Previous stroke | 1.632 (1.088-2.448) | 0.0179 |  | |  |
| Atrial fibrillation | 1.497 (1.043-2.148) | 0.0286 |  | |  |
| Dementia | 1.349 (0.585-3.111) | 0.4824 |  | |  |
| Depression | 0.815 (0.241-2.750) | 0.7414 |  | |  |
| Pulmonary disease | 1.349 (0.585-3.111) | 0.4824 |  | |  |
| Ulcerative disease | 3.827 (1.839-7.966) | 0.0003 | 2.415 (1.046-5.574) | | 0.0388 |
| Liver disease | 2.269 (0.879-5.857) | 0.0903 |  | |  |
| Renal disease | 2.704 (1.470-4.974) | 0.0014 |  | |  |
| Connective tissue disease | 0.479 (0.021-10.735) | 0.6427 |  | |  |
| Diabetes | 3.076 (2.117-4.470) | <.0001 | 2.706 (1.816-4.030) | | <.0001 |
| Anemia | 2.466 (1.708-3.562) | <.0001 | 1.770 (1.176-2.664) | | 0.0062 |
| Cancer | 1.659 (0.857-3.211) | 0.1332 |  | |  |
| Metastatic cancer | 14.874 (5.555-39.825) | <.0001 | 11.239 (2.987-42.282) | | <.0001 |

OR, odds ratio; NIHSS, National Institutes of Health Stroke Scale.

**Supplemental Table 6. Comparison of scoring system in reperfusion therapy**

|  | Equation |  | AUC |
| --- | --- | --- | --- |
| PRE score^1^ | age (years)+2×NIHSS score −10×ASPECTS | EVT | 0.79 |
| SPAN index^2^ | Age + NIHSS score | IVT | 0.64 |
| THRIVE score^3^ | 1 point for age 60 to 79 years,  2 points for age ≥80 years,  2 points for NIHSS score 11 to 20,  4 points for a NIHSS score ≥21,  1 point each for hypertension, diabetes mellitus, and atrial ﬁbrillation  Range 0 to 9 | EVT | 0.709 |
| HIAT score^4^ | 1 point for age 75 years;  1 for NIHSS score >18  1 point for glucose 150 mg/dL (range, 0 to 3 mg/dL)  Range 0 to 3 | EVT | 0.73 |
| HIAT2 score^5^ | age (≤59=0, 60–79=2, ≥80 years=4),  glucose (<150=0, ≥150=1),  NIHSS score (≤10=0, 11–20=1, ≥21=2),  ASPECTS (8–10=0, ≤7=3).  Range 0 to 10 | EVT | 0.748 |
| Current reperfusion comorbidity index | **Without considering NIHSS score**  predicted probability = 1 / (1 + exp [- A]), where A = - 5.2999 + 0.0357 × age + 0.6666 × myocardial infarction + 0.6622 × congestive heart failure + 1.0187 × ulcer disease + 1.0201 × diabetes mellitus + 0.5568 × anemia + 2.8128 × metastatic cancer.  **With considering NIHSS score**  predicted probability = 1 / (1 + exp [- A]), where A = -6.2807 + 0.0307 x age + 0.1019 x NIHSS score + 0.8817 x ulcer disease + 0.571 x anemia + 0.9953 x diabetes mellitus + 2.4194 x metastatic cancer. | IVT and EVT | 0.747  0.781 |

Pittsburgh Response to Endovascular Therapy (PRE) score; the Stroke Prognostication Using Age and National Institutes of Health Stroke Scale (SPAN) index; the Totaled Health Risks in Vascular Events (THRIVE) score; the Houston Intra-Arterial Therapy (HIAT) score; NIHSS, National Institutes of Health Stroke Scale; ASPECTS, The Alberta Stroke Program Early CT Score; IVT, intravenous thrombolysis; EVT, endovascular thrombectomy.

**Supplemental Figure 1. Receiver-operating characteristic curve (ROC) comparison death within 6 months mortality considering initial stroke severity (A) and external validation (B)**

ROC comparison for death within 6 months showed that the AUC did not differ between the reperfusion comorbidity considering initial stroke severity (age, NIHSS score + 4 comorbidities [ulcer disease, diabetes mellitus, anemia, and metastatic cancer]) and the original CCI (age, NIHSS score + 16 comorbidities) (p = 0.120) (A). In the prospective cohort, the predictive ability of the reperfusion comorbidity index was AUC of 0.821 (95% CI, 0.759 - 0.884) (B).

**
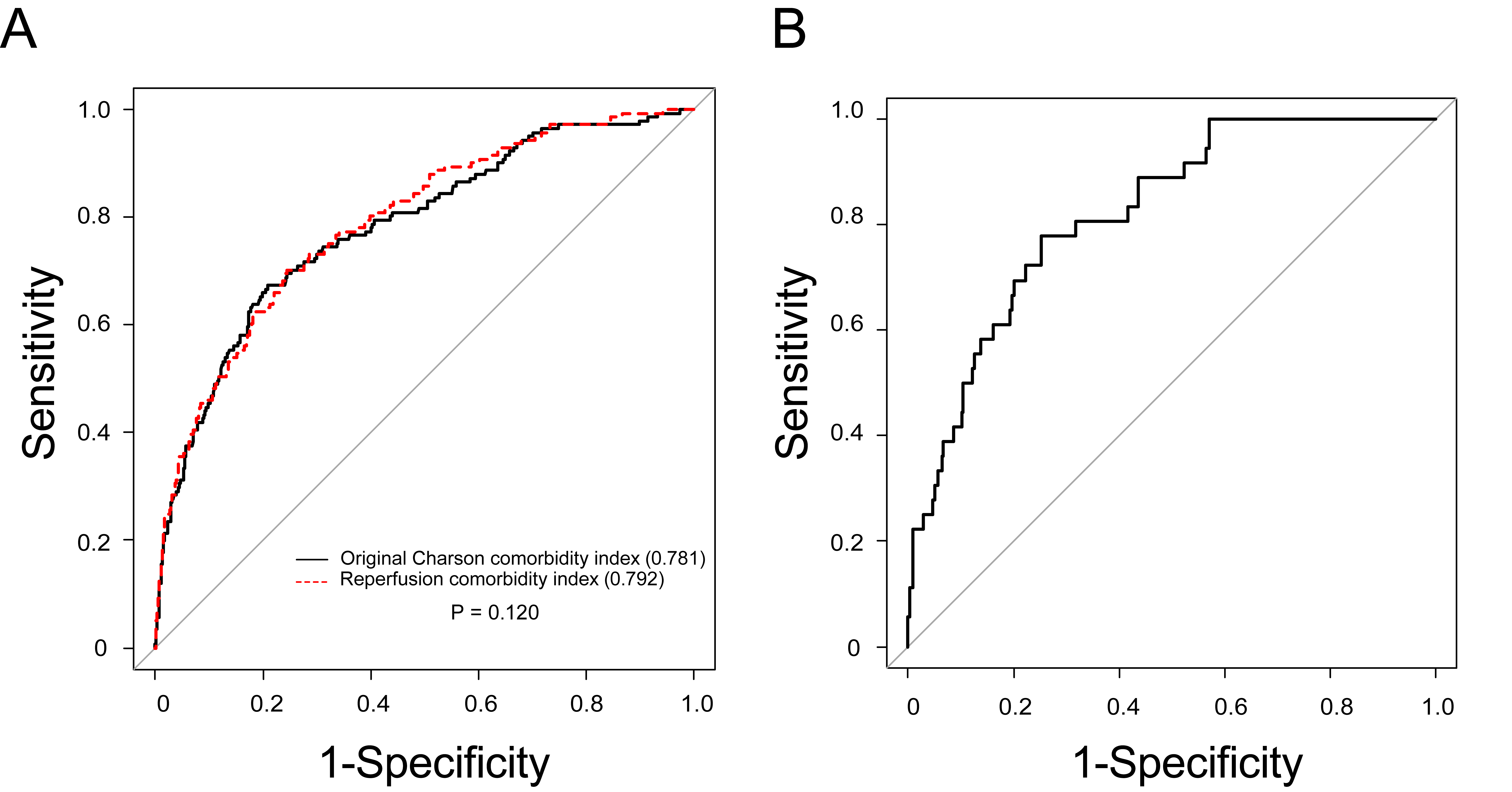
**

# Supplemental material. Definition of comorbidities

1. Myocardial infarction: A diagnosis of myocardial infarction was made within 6 months before admission. These patients should have been hospitalized for chest pain or an equivalent clinical event and have had electrocardiographic and/or enzyme changes. Patients with electrocardiographic changes alone who have no clinical history are not designated as having had an infarction.

2. Congestive heart failure: Congestive heart failure includes patients who have had exertional or paroxysmal nocturnal dyspnea and who have responded symptomatically to digitalis, diuretics, or afterload reducing agents. It does not include patients who are on one of those medications but who have had no response and no evidence of improvement of physical signs with treatment.

3. Peripheral vascular disease: Peripheral vascular includes patients with intermittent claudication or those who had a bypass for arterial insufficiency, those with gangrene or acute arterial insufficiency, and those with a treated or untreated thoracic or abdominal aneurysm (6 cm or more).

4. Previous stroke: Previous stroke includes patients with a history of a cerebrovascular disease or transient ischemic attacks before admission.

5. Atrial fibrillation: A diagnosis of atrial fibrillation was made by electrocardiography or Holter monitoring before or after admission.

6. Dementia: A diagnosis of dementia was made according to the Diagnostic and Statistical Manual of Mental Disorders (DSM) IV criteria or patient have a previous history of dementia diagnosis.

7. Depression: Depression includes patients who are currently receiving treatment for depression, whether pharmacologic or psychotherapy, or cognitive behavioral therapy. A diagnosis of depression was made 6 months before admission.

8. Chronic pulmonary disease : Chronic pulmonary disease includes patients with asthma, chronic bronchitis, emphysema, and chronic obstructive pulmonary disease.

9. Ulcer disease: Peptic ulcer disease includes patients who have required treatment for ulcer disease, including those who have bled from ulcers 6 months before admission.

10. Mild liver disease: Mild liver disease consists of chronic hepatitis(B or C)or cirrhosis without portal hypertension.

11. Moderate or severe renal disease: Moderate renal insufficiency includes patients with a serum creatinine >3mg/dl. Severe renal disease includes patients on dialysis, those who had a transplant, and those with uremia.

12. Connective tissue disease: Rheumatologic disease includes patients with systemic lupus erythematous, polymyositis, mixed connective tissue disease, rheumatoid arthritis, polymyositis, polymyalgia rheumatic, vasculitis, sarcoidosis, Sjögren syndrome or any other systemic vasculitis.

13. Anemia: Anemia includes hemoglobin level at admission is reduced (Hgb ≤13 in men, Hgb ≤12 in women).

14. Diabetes: A diagnosis of diabetes was made before or after admission or includes patients with diabetes treated with insulin or oral hypoglycemic, but not diet alone. Diabetes during pregnancy alone is not counted.

16. Acquired immune deficiency syndrome (AIDS): AIDS includes patients with definite or probable AIDS.

17. Non-metastatic solid tumor: Non-metastatic solid tumor consists of patients with solid tumors without documented metastases, including breast, colon, lung, prostate, and variety of other tumors. The patient was included when the patient diagnosed or treated for malignancy within 6 months.

18. Leukemia: Leukemia includes patients with acute and chronic myelogenous leukemia. Acute and chronic lymphocytic leukemia, and polycythemia vera.

The patient was included when the patient diagnosed or treated for malignancy within 6 months.

19. Lymphoma: Lymphoma includes patients with Hodgkins, lymphosarcoma, Waldenstrom’s macroglobulinemia, myeloma, and other lymphomas. The patient was included when the patient diagnosed or treated for malignancy within 6 months.

20. Metastatic cancer: Metastatic cancer includes patients with metastatic solid tumors, including breast, lung, colon and other tumors. The patient was included when the patient diagnosed or treated for malignancy within 6 months.
